# Intrasphenoidal Rathke’s Cleft Cyst: An Uncommon Feat

**DOI:** 10.7759/cureus.33206

**Published:** 2023-01-01

**Authors:** André De Sousa Machado, Ana Silva, Jose Silva, José R Brandão, Luís Meireles

**Affiliations:** 1 Otolaryngology - Head and Neck Surgery, Centro Hospitalar Universitário do Porto, Porto, PRT; 2 Neuroradiology, Centro Hospitalar Universitário do Porto, Porto, PRT; 3 Pathology, Centro Hospitalar Universitário do Porto, Porto, PRT

**Keywords:** sphenoid sinus lesions, endoscopic approach, rathke, endoscopic transnasal approach, rathke's cleft cyst

## Abstract

Usually occurring entirely intrasellarly or extending suprasellarly (intra-suprasellar), Rathke’s cleft cysts (RCCs) can present with an intrasphenoidal location. Extrasellar positions are rare. To date, only seven patients with intrasphenoidal RCC have been reported in the literature. Despite the rarity of the condition and the lack of pathognomonic radiological features, preoperative diagnosis remains challenging. A trans-sphenoidal approach can be adopted to treat this type of cyst, which has great clinical relevance. Awareness of this different presentation of RCC before respective management may be of value in its approach. Intrasphenoidal RCC should be diagnosed preoperatively and the surgical approach should be changed accordingly by aspiration and partial removal before the histological examination.

## Introduction

Rathke’s cleft cysts (RCCs) in the nasopharyngeal region are midline cystic lesions with rare incidence [1]. No pathognomonic clinical and radiologic features are usually found, although, the awareness of this kind of presentation as we can adjust the surgical management of the cyst in a trans-sphenoidal approach is of great clinical relevance. RCC treatment is different from other lesions found in this region, namely craniopharyngioma or sphenoidal mucocele [2].

## Case presentation

**Figure 1:**
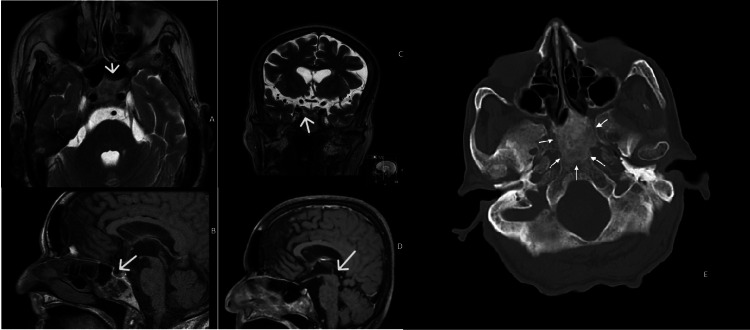
Axial (A) and coronal (B) T2-weighted images, and sagittal T1-weighted images before (C) and after (D) contrast administration. The lesion has a heterogeneous signal on spontaneous T1 (C) and T2 images (A and B) – it is slightly hyperintense on T2 and markedly hyperintense on T1 peripherally, and iso/hypointense on T2 and hypointense on T1 centrally. On postcontrast images, there is a diffuse enhancement (D). Axial bone window CT image showing an ill-defined ground-glass density lesion centered in the clivus, with loss of normal bone cortication; The lesion has a maximum antero-posterior extension of 35mm, but there is no significant mass effect. (1-E)

A woman in her 70s presented with progressive headaches for three months, with intensity on a Visual Analog Scale of 5 out of 10. These headaches were persistent throughout the day, located in the parietal-frontal region with no irradiation to other regions. In terms of aggravating factors, the patient complained of an increase in pain with cough and defecation. The patient took paracetamol 500mg, twice a day for one week, with no relief of the symptoms, and referred no alleviating factors. Patient had a history of Pott disease with paraplegia for 50 years and obesity with a BMI of 35. Blood pressure was 140/56mmHg with a heart rate of 72 bpm on average. She sought primary care who referred her to a Neurology specialist who, in turn, requested a CT scan. This CT scan showed a lesion in the sphenoidal region. There were no visual symptoms, body temperature deregulation, loss of libido, changes in urination habits. Cranial pair assessment was unremarkable. Neurologic exam was also unremarkable except for paraparesis of inferior limbs. The patient was promptly referred to the ENT department after hormonal study and ophthalmologic examination at the Neurology department. From the onset of symptoms to the diagnosis of this mass, four months passed. After a detection of the mass on MRI, two months were needed to reach a consensus to its surgical excision. Flexible fibroscopy and rigid rhinoscopy were performed at the ENT department. There were no other findings on physical examination. The patient's ophthalmologic examination and hormone study were normal - pituitary hormones, adrenal cortical hormones, thyroid hormones and hypothalamic releasing hormones values were at the range of the levels of reference. During the follow-up no change of size of the mass was seen. At the ENT evaluation, rigid rhinoscopy of 0, 30 and 70 degrees and flexible fibroscopy revealed hypertrophy of the inferior turbinates with no accessory visible masses. CT scan demonstrated a lesion of the sphenoidal region by low-attenuation cystic mass without calcification. MRI imaging showed a cystic sphenoidal extradural lesion, isointense on T1-weighted images (WI) with peripheral gadolinium enhancement and hyperintense on T2 WI (Figure 1).

**Figure 2:**
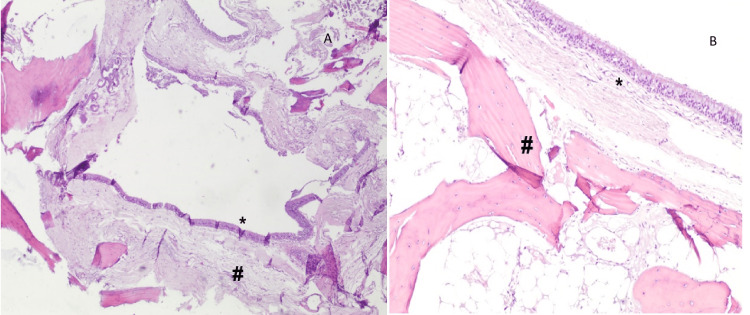
Cystic lesion surrounded by bone tissue and lined by respiratory epithelium with no atypia, consistent with the diagnosis. Respiratory-type epithelium lining cyst (*), surrounded by mature osseous bone (#). A: 40x, B: 100x

The differential diagnoses included craniopharyngioma and mucocele. A bulge was identified in the posterior wall of the sphenoid sinus by an endoscopic transnasal approach. As soon as the region was reached and penetrated, yellow globules of tissue and a mucous-like fluid began to drain. Histopathological examination was also conducted on part of the cyst wall (Figure 2).

**Figure 3:**
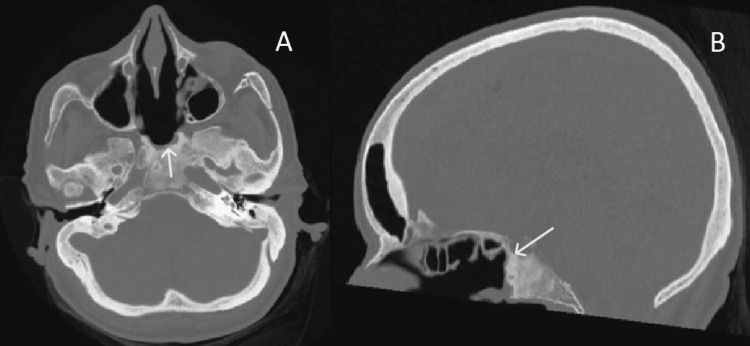
Axial (A) and sagittal (B) bone window CT images of the same patient, after endoscopic surgical approach. There was partial removal of the lesion.

The cyst wall was thick and hard to penetrate, with a partial evacuation following excision with marsupialization of the mass. On microscopy, fragments of mature osseous trabeculae are seen surrounding a cystic lesion, which is lined by ciliated pseudostratified columnar epithelium with mucinous goblet cells (respiratory-type epithelium). The postoperative course was uneventful and patient was discharged home after two days. Headaches disappeared after one week, and the patient didn’t develop neurologic symptoms or post-operative complications from the endoscopic surgery (Figure 3).

At present time and after nine-month follow-up, no recurrence has been verified. Until the day, a follow-up is being maintained under a close surveillance of the symptoms and a MRI yearly in the first five years, and every two years after that.

## Discussion

RCCs have their origin in Rathke’s pouch. They appear mostly within the sella turcica, eventually extending to the suprasellar region [1-3]. Its pathogenesis can be linked with its origin and this may explain its intrasellar location [3]. Although commonly asymptomatic, an RCC with symptoms usually presents in adulthood with headache, endocrine dysfunction (hyperprolactinemia or growth hormone deficiency, or even diabetes insipidus) and visual loss, this being the most common symptom [4-6]. Intrasphenoidal RCC is a different pathology when compared to other RCC locations; it doesn't not present with visual or endocrine pathology. To date, seven patients have been reported in the literature, four of them presented with diplopia, probably due to the extension of the lesion to the middle fossa and orbit, and one was asymptomatic [7-9] (Table 1).

**Table 1:** Summary of the intrasphenoidal Rathke’s cleft cyst patients reported in the literature

Study	Age (years)/ gender	Diagnosis	Size	Symptoms	Treatment	Post-operative complications	Follow-up duration	Recurrence
Pojskic [10]	65/ female	Intrasphenoidal Rathke Cleft Cyst and Silent ACTH Adenoma	Not mentioned	Headache and nasal stuffiness	Transnasal endoscopic surgery	Uneventful	6 months	No
Kasliwal [9]	21/ male	Intrasphenoidal Rathke Cleft Cyst	4cm	Diplopia, headache	Endoscopic drainage and partial resection	Uneventful	2 years	No
Kocaman [11]	28/ female	Intrasphenoidal Rathke Cleft Cyst	Not mentioned	Headache extending to the left eye	Transnasal transsphenoidal endoscopic surgery	Not mentioned	1 year	No
Kalina [8]	13/male	Intrasphenoidal Rathke Cleft Cyst	Not mentioned	Suspected seizure	Transnasal endoscopic surgery	Uneventful	3-months	No
Nagarajan [12]	15/ female	Intrasphenoidal Rathke Cleft Cyst	Not mentioned	Headache, vomiting and diplopia	Trans-nasal endoscopic trans-sphenoidal decompression	No	18-months	No
Megdiche-Bazarbacha [13]	41/male	Intrasphenoidal Rathke Cleft Cyst	4cm	Frontal headaches, loss of visual acuity on left eye, ptosis and diplopia	Surgery – transrhinoseptal approach	Uneventful	1 year	No
Hamouda [4]	41/male	Sphenoidal and nasopharyngeal Rathke cleft cyst	4cm	Headaches, loss of visual acuity on left eye, ptosis and diplopia	Surgery – transrhinoseptal approach	Uneventful	1 year	No recurrence of the sphenoidal cyst; persistence of the lesion of the nasopharynx.

MRI presents of utmost importance in the preoperative assessment for differential diagnosis of RCC with other cystic sellar lesions. Usually, RCCs are well-defined with a homogeneous appearance. More than 60% of the lesions are hyperintense on T1-MRI, because of the presence of mucous. On T2-weighted MRI, the lesions are mostly hyperintense, while 33% can be iso- to hypointense. Often, contrast reinforcement is missing, and a thin magnifying edge is found - usually credited to inflammation or squamous metaplasia of the cyst wall or to a circumferential rim of the displaced pituitary gland [7,14,15]. This enhancing rim is often considered pathognomonic and referred to as the claw sign. RCC frequently goes undiagnosed preoperatively, often mistaken as cystic craniopharyngioma, which is more prevalent. A preoperative diagnosis is of great importance; aspiration and marsupialization of an RCC by an endonasal trans-sphenoidal route are enough as these cysts are benign with the respective cyst sheath presenting a low proliferating activity. Histopathological analysis is the gold standard for the respective diagnosis. On microscopy, fragments of mature osseous trabeculae are seen surrounding a cystic lesion, which is lined by ciliated pseudostratified columnar epithelium with mucinous goblet cells (respiratory-type epithelium). The presence of squamous metaplasia or stratified squamous epithelium, seen in some cases but not in ours, has been linked with a higher recurrence of the pathology [16]. No content could be identified in the histological sample and, also, there was no significant evidence of inflammation, cholesterol granulomas nor xanthomatous cells in the fibrous wall of the cyst. Histopathological study should be oriented for RCC as cystic lesions of sellar and parasellar regions can have similar features and eventually be misdiagnosed if not taken into account [14-16]. Close communication between the ENT surgeon, radiologist, neurosurgeon, and pathologist remains vital due to the unusual site of the pathology. The approach of RCC changed over time [7,8,14,17]. A conservative approach of RCC for those which are discovered incidentally is advocated, although with clinical and radiological follow-up. Surgical drainage via transnasal transsphenoidal approach is the modality of choice in patients who present visual field deficits or underlying laboratory evidence of endocrinopathy [6,7].

Generally speaking, recurrence rates after surgical approach of RCC can assume values up to one-third of cases - being correlated with factors such as the shape of enhancement of the cyst wall, existence of squamous metaplasia, signs of chronic inflammation or stratified epithelium, fierceness of cyst wall resection, and the use of fat graft in an eventual repair [6,7]. When compared to RCC, the intrasphenoidal subtype does not seem to present different clinical features that can easier the pre-operative diagnostic process [9-13]. As RCC presents in the posterior wall of the sphenoid sinus, usually this cannot be properly evaluated in the fibroscopy, except if there was a previous intervention in this area with opening of the anterior portion of the sphenoid sinus [18]. Although there is not a single tool to evaluate patient-reported outcomes in patients with sinonasal tumors, the literature refers that a combination of a nasal-specific tool as SNOT-22, and health outcome measures such as Short Form health survey questionnaire (SF-36) and Karnofsky Performance Status can give more perspective into the quality of life of patients with nasal and paranasal tumors and its course after surgery [19]. Even though it was not performed at the time of the surgery that can be pointed out as a limitation we consider of utmost importance in the respective management of these pathologies.

## Conclusions

Intrasphenoidal RCC is a midline cystic lesion and a rare entity. Biopsy of the lesion and respective histopathology is the cornerstone of diagnosis. Awareness of this different presentation of RCC before respective management may be of value in its approach. Intrasphenoidal RCC should be diagnosed preoperatively and the surgical approach should be changed accordingly by aspiration and partial removal before the histological examination.
